# Is synthetic data generation effective in maintaining clinical biomarkers? Investigating diffusion models across diverse imaging modalities

**DOI:** 10.3389/frai.2024.1454441

**Published:** 2025-01-31

**Authors:** Abdullah Hosseini, Ahmed Serag

**Affiliations:** AI Innovation Lab, Weill Cornell Medicine-Qatar, Doha, Qatar

**Keywords:** synthetic data generation, clinical biomarkers, denoising diffusion models, medical imaging, Swin-transformer network

## Abstract

**Introduction:**

The integration of recent technologies in medical imaging has become a cornerstone of modern healthcare, facilitating detailed analysis of internal anatomy and pathology. Traditional methods, however, often grapple with data-sharing restrictions due to privacy concerns. Emerging techniques in artificial intelligence offer innovative solutions to overcome these constraints, with synthetic data generation enabling the creation of realistic medical imaging datasets, but the preservation of critical hidden medical biomarkers is an open question.

**Methods:**

This study employs state-of-the-art Denoising Diffusion Probabilistic Models integrated with a Swin-transformer-based network to generate synthetic medical data. Three distinct areas of medical imaging - radiology, ophthalmology, and histopathology - are explored. The quality of synthetic images is evaluated through a classifier trained to identify the preservation of medical biomarkers.

**Results:**

The diffusion model effectively preserves key medical features, such as lung markings and retinal abnormalities, producing synthetic images closely resembling real data. Classifier performance demonstrates the reliability of synthetic data for downstream tasks, with F1 and AUC reaching 0.8–0.99.

**Discussion:**

This work provides valuable insights into the potential of diffusion-based models for generating realistic, biomarker-preserving synthetic images across various medical imaging modalities. These findings highlight the potential of synthetic data to address challenges such as data scarcity and privacy concerns in clinical practice, research, and education.

## 1 Introduction

Medical imaging serves as a cornerstone of modern medicine, offering indispensable insights into the human body’s internal structures and physiological processes (Jha et al., 2020; Mazurowski et al., 2023; Veta et al., 2014). Over the years, imaging biomarkers have emerged as crucial indicators for diagnosing diseases, monitoring treatment efficacy, and understanding disease progression (Yan et al., 2023; Liu et al., 2024; Moor et al., 2023; Ronneberger et al., 2015). While traditional imaging techniques excel at capturing these biomarkers, they often present challenges such as high costs, limited accessibility, and concerns surrounding data security and patient privacy, which can hinder the sharing of medical imaging datasets (Mittelstadt and Floridi, 2016). Moreover, in cases where the incidence rate of a disease is low, the process of accumulating a sufficiently sized dataset can span years. These limitations have spurred significant interest in AI-driven approaches, with a particular focus on synthetic image generation as a means to overcome these challenges. Recent studies have highlighted the potential of artificial intelligence (AI) methodologies to produce synthetic images that not only mimic the visual characteristics of real medical images but also preserve the subtle imaging biomarkers essential for accurate clinical interpretation and disease classification (Fan et al., 2024; Zhang et al., 2023; Thambawita et al., 2022; Thakur and Thakur, 2024; Pan et al., 2023).

The generation of synthetic medical images poses a unique set of challenges compared to other domains due to the intricate nature of biological structures and the subtle nuances of imaging biomarkers (Perez et al., 2024). While existing AI approaches have demonstrated remarkable capabilities in generating realistic images, preserving the underlying biomarkers remains a significant hurdle. Without faithful representation of these biomarkers, synthetic images risk being of limited utility in clinical settings, hindering their adoption for tasks such as training AI models, augmenting datasets, and validating imaging algorithms.

In this paper, we explore an innovative AI methodology specifically tailored for generating synthetic medical images while preserving concealed imaging biomarkers. Our approach builds upon recent advancements in deep learning (DL), leveraging denoising diffusion probabilistic model (DDPM) (Ho et al., 2020) to produce high-fidelity images that not only resemble real medical scans but also retain the critical biomarkers necessary for accurate clinical interpretation. By integrating domain-specific knowledge and data-driven learning, our work aims to bridge the gap between synthetic and real-world medical images, offering a promising solution to the challenges associated with imaging biomarker preservation.

The aim of current research is to explore the feasibility of generative AI diffusion models for generating realistic, high-quality synthetic medical data while preserving medical biomarkers and statistical properties of the original data. This approach could facilitate data sharing without compromising patient confidentiality, thereby aiding in the training and testing of new AI systems for disease diagnosis and classification. Our key contributions: (1) the utilization of a state-of-the-art diffusion-based model for generating conditional images across diverse medical imaging modalities, including radiology, ophthalmology and histopathology; (2) empirical evidence showcasing that features extracted from the synthesized data closely resemble those derived from real data; (3) experimental validation showcasing that generated images effectively preserve important medical biomarkers, by solely using the synthesized data to train classifiers for disease diagnoses and testing it on unseen real datasets.

## 2 Methods

### 2.1 Dataset and preprocessing

In this work, three distinct datasets, from the fields of radiology, ophthalmology, and histopathology, were utilized. From radiology, we used a chest X-ray dataset (Kermany et al., 2018), designed for the detection of healthy X-ray images from those depicting Pneumonia disease. This dataset consists of 5,840 grayscale images, each with a resolution of 256 × 256 pixels. A fold constituting 10 percent of the dataset is allocated specifically for testing purposes.

The second dataset utilized is an ophthalmology OCT dataset (Kermany et al., 2018), designed for multiclass classification tasks aimed at detecting four distinct categories within OCT images. These classes comprise Choroidal Neovascularization (CNV), Diabetic Macular Edema (DME), drusen, and normal retina with the approximate size of 37,200, 11,300, 8,600, and 26,300 images, respectively. For testing, we followed the dataset structure, allocating 242 images per class as unseen data, while the remaining dataset was used for training. To ensure uniformity in image dimensions for training purposes, a pre-processing step was implemented wherein images with variant slice shapes were padded by black background pixels, resulting in a standardized size of 512 × 512 pixels.

The third dataset is a publicly available histopathology dataset (Janowczyk and Madabhushi, 2016; Cruz-Roa et al., 2014), aimed at classifying patches as either Invasive Ductal Carcinoma (IDC), the most common subtype of all breast cancers, or as healthy tissue. The original dataset comprises 162 whole mount slide images of Breast Cancer (BCa) specimens scanned at 40x magnification. From these images, 277,524 patches of size 50 × 50 pixels were extracted, consisting of 198,738 IDC-negative patches and 78,786 IDC-positive patches. For training the diffusion models, only 22,000 patches were utilized, with the remaining patches allocated for testing purposes.

### 2.2 Denoising diffusions models

Denoising Diffusion Probabilistic Model (DDPM) (Ho et al., 2020), were employed to generate high-fidelity synthetic medical images. These models transform Gaussian noise into synthetic images through a diffusion process (Dhariwal and Nichol, 2021; Nichol and Dhariwal, 2021). The process begins with the transformation of a two-dimensional Gaussian noise sample, $X_{t}\sim N\left(0,1\right)$ , into a synthetic image, *x*, by means of a diffusion procedure. This diffusion method involves incrementally adding a small noise component, *ε*, onto the image *x* over *t* timesteps, gradually converting *x* into a pure Gaussian noise sample *T*; this transformation is typically referred to as the forward diffusion process. Subsequently, the Gaussian noise sample T is restored to its original, noise-free form, *x*, by systematically removing the added noise, *ε*, in a process known as reverse diffusion. In this work, we incorporated Swin-Vision transformers as the denoising function estimator proposed by Pan et al. (2023), ensuring high performance across modalities.

### 2.3 Image quality evaluation metrics

To evaluate the quality of synthetic images in comparison to real images, particularly in terms of structural fidelity, diversity, and distributional similarity, we employed three widely used metrics: the Inception Score (Salimans et al., 2016), Fréchet Inception Distance (FID) (Heusel et al., 2017), and nearest Structural Similarity Index Measure (SSIM).

The Inception Score was utilized to quantitatively assess the diversity and visual quality of the synthetic images, as it reflects how closely the synthetic images resemble the distribution of real images in the feature space. A higher IS indicates greater quality and diversity of the generated images. FID score was employed as a measure of fidelity, capturing the similarity between the distributions of features extracted from real and synthetic images. A lower FID score indicates a closer match between the two distributions, signifying higher quality and realism in the synthetic images.

To assess structural similarity, each synthetic image was compared against all other synthetic images using the Structural Similarity Index Measure (SSIM). From these comparisons, the closest matching pair was identified for each image. The SSIM values of these closest pairs were aggregated and reported as the nearest SSIMs for the synthetic dataset. The rationale behind this approach is that the nearest SSIM values of real images tend to cluster within a characteristic range, and synthetic images are expected to exhibit a comparable distribution of nearest SSIM values, thereby demonstrating structural consistency across the datasets.

Additionally, to evaluate the similarity between synthetic and real images, we utilized baseline pretrained models from MedMNIST (Yang et al., 2023) for colon classification (used for Breast Histopathology), pneumonia classification, and OCT classification. These pretrained models were employed to extract feature embeddings from the images, which were subsequently visualized using t-SNE. Directly applying t-SNE to the raw image pixels was avoided due to the computational inefficiency associated with high-dimensional data processing. By leveraging pretrained models, the images were compressed into lower-dimensional embeddings, reducing computational costs and providing a more meaningful representation of the data. Since the pretrained models were trained on real medical image classification tasks, they capture clinically relevant and semantically rich features. This approach facilitated a robust comparison between real and synthetic images, as the extracted embeddings encapsulate higher-level features rather than pixel-level similarities. Consequently, the use of pretrained models enabled us to assess the extent to which the generated synthetic images preserved critical characteristics of real images, offering deeper insights into their quality and fidelity.

### 2.4 Evaluation of medical imaging biomarker fidelity in synthetic images using classification tasks

**Figure 1:**
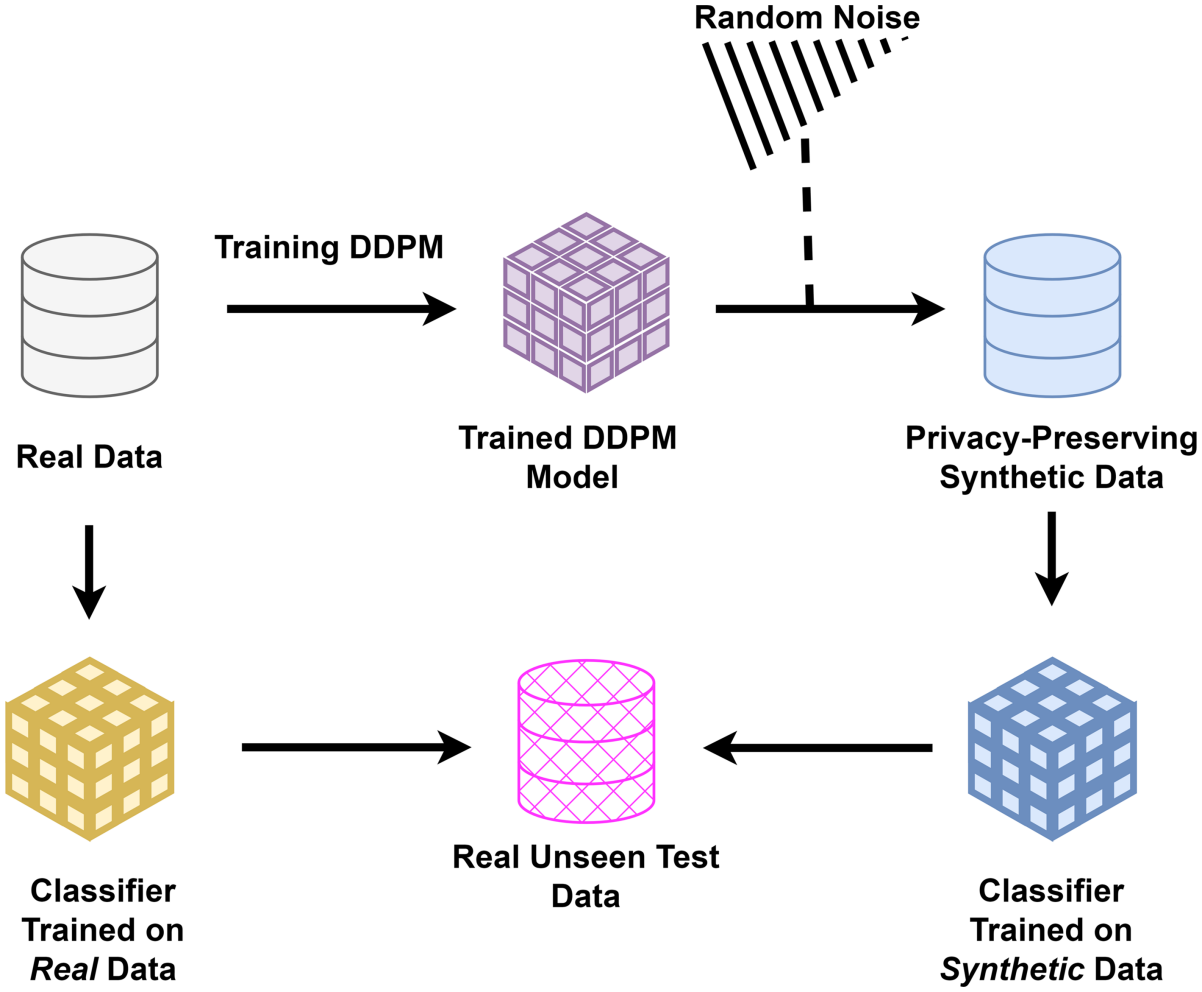
Study architecture for evaluating synthetic data: classifiers were trained separately on real and synthetic medical images generated using DDPM. Both were tested on unseen real data to assess the preservation of diagnostic biomarkers and the privacy-preserving potential of synthetic images in healthcare applications.

To evaluate the preservation of diagnostic biomarkers, classifiers were trained separately on real and synthetic data. This step was critical to demonstrate that synthetic data could replace real data in sensitive applications without compromising privacy or diagnostic accuracy. For each imaging modality and dataset, two separate classifiers were trained: one using real images and the other exclusively using synthetic images. Both classifiers for each modality were subsequently tested on an identical, unseen test set comprising real images, see Figure 1. For each modality, we utilized widely recognized classifier architectures in our evaluations (Simonyan and Zisserman, 2014). The performance of the classifiers trained on real and synthetic images, respectively, was then assessed using metrics including Area Under the Curve (AUC), precision, recall, and accuracy.

### 2.5 Statistical analysis

To test for differences between classification results, *t*-tests were used for normally distributed data, and Mann Whitney U was used to compare non-normal distributions (Shapiro–Wilk normality test was used). *p* < 0.05 were considered significant after controlling for error using false discovery rate (FDR).

## 3 Results

The performance of the DDPM model has been assessed in two key aspects: first, by visually analyzing the similarity of generated images to gauge their resemblance to real images. Second, by training a classifier on synthetic data to classify diseases and subsequently testing it on unseen data to determine whether synthetic medical images can capture medical imaging biomarkers.

### 3.1 Visual inspection of diffusion model-generated medical images

**Figure 2:**
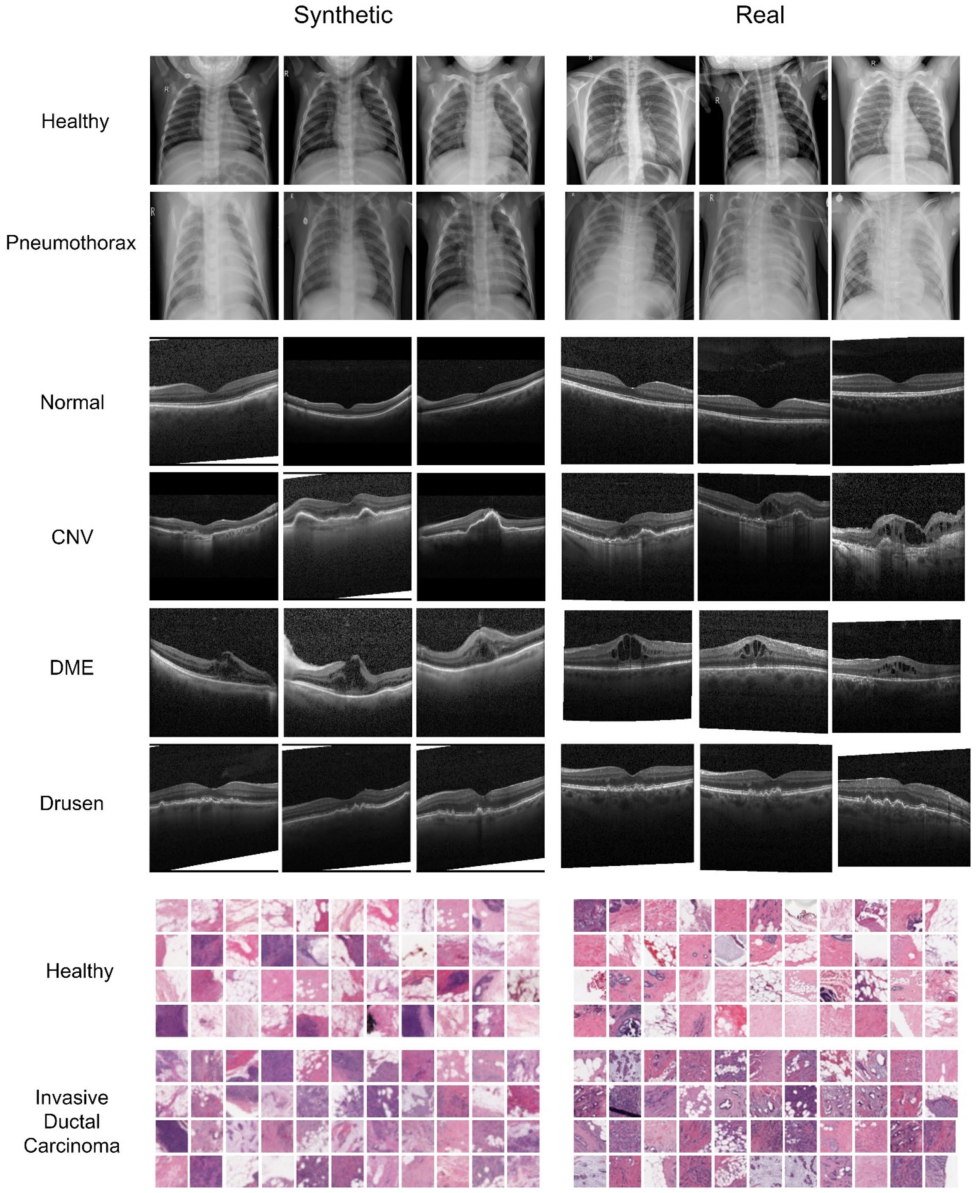
Comparison of synthesized and real images from the Chest X-ray, OCT, and Breast Cancer Histopathology datasets. The first two rows depict healthy and Pneumonia chest X-ray images, respectively, each resized to 256 × 256 pixels. The following four rows represent various classes of OCT images. In the histopathological images, the last two rows illustrate healthy and unhealthy patches, respectively, with each breast cancer histopathology patch measuring 32 × 32 pixels.

Figure 2 showcases examples of Chest X-ray, OCT, and breast cancer histopathology images generated by the diffusion model. In the Chest X-ray samples, discernible characteristics such as linear shadow reduced lung markings surrounding it, indicative of a collapsed lung, are prominently captured by the diffusion model and faithfully preserved in the generated image variations (Huan et al., 2021). Regarding OCT image analysis, DME manifests as generalized retinal swelling, discernible in both synthetic and authentic datasets. Conversely, CNV lesions tend to exhibit larger dimensions, especially in width, as observed on OCT scans. Moreover, Drusen are recognized as distinct bumps or deposits situated beneath the retina, a characteristic apparent in both synthetic and real data (Cheung et al., 2017; Spaide and Curcio, 2010; Otani et al., 1999).

### 3.2 Quantitative evaluation of synthetic medical images

**Figure 3:**
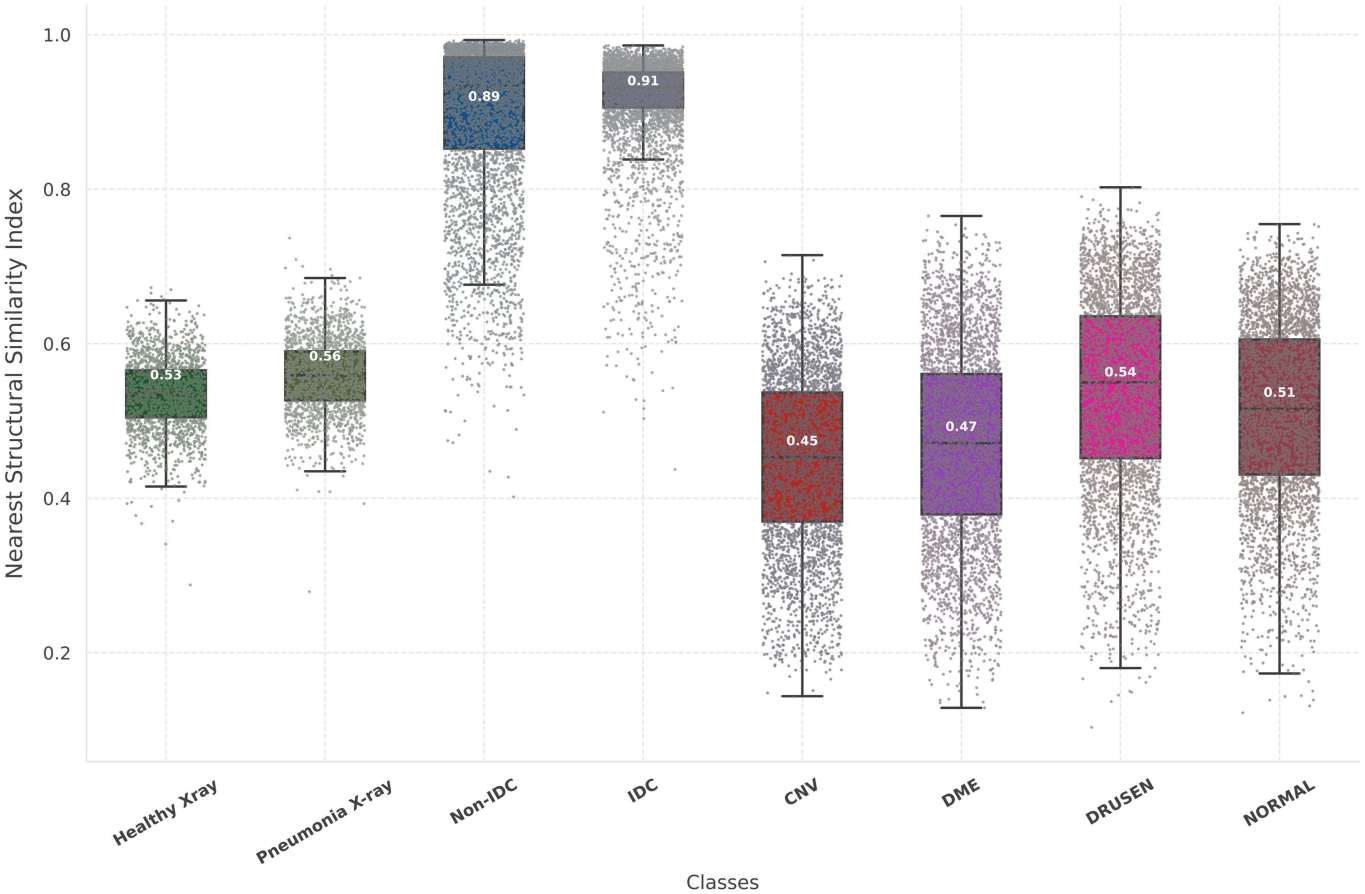
Illustration of the nearest SSIM values between each synthetic image and its closest matching pair, where lower values indicate better model performance. The nearest SSIM values are shown for real images and synthetic images across three datasets: chest X-rays (first two box plots), breast cancer histopathology (third and fourth box plots), and OCT images (last four box plots). Each box plot includes colored dots that depict the SSIM values for all sampled.

The Inception scores for the Chest X-ray, OCT, and breast cancer histopathology dataset are 2.45, 2.05, and 3.28, respectively. For the Chest X-ray dataset, FID scores are 46.76 for healthy images and 44.64 for unhealthy images (representing Pneumonia), indicating a close resemblance between real and synthetic images. In the OCT images, the average FID score across four classes is 81.83, DME presenting the most challenging class with an FID of 102.13. In contrast, Drusen emerges as the class closest to real images, boasting an FID score of 53.53. For the breast cancer histopathology dataset, FID scores are notably higher at 106.69 for healthy images and 109.97 for unhealthy images (representing IDC), suggesting a somewhat greater disparity between real and synthetic distributions compared to the Chest X-ray dataset. Figure 3 provides a detailed and comprehensive analysis of the nearest SSIM values, broken down by each class within the synthetic dataset, offering further insights into the structural fidelity of the generated images. It is important to note that, due to the large size of the breast cancer histopathology and OCT datasets, 5,000 random samples were selected for each class within these datasets to ensure a representative analysis.

### 3.3 Feature comparison and statistical analysis of synthetic and real medical images

**Figure 4:**
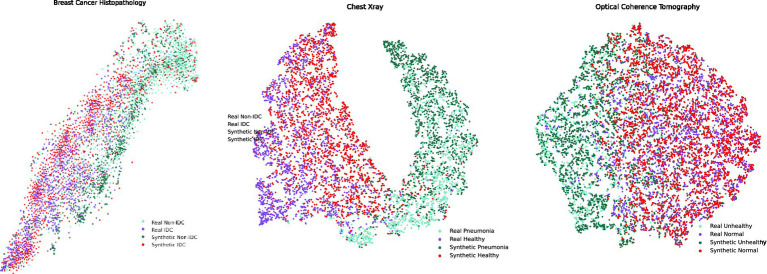
t-SNE feature space visualization of synthetic images generated by MT-DDPM for the Chest X-ray and Breast Cancer Histopathology datasets.

**Figure 5:**
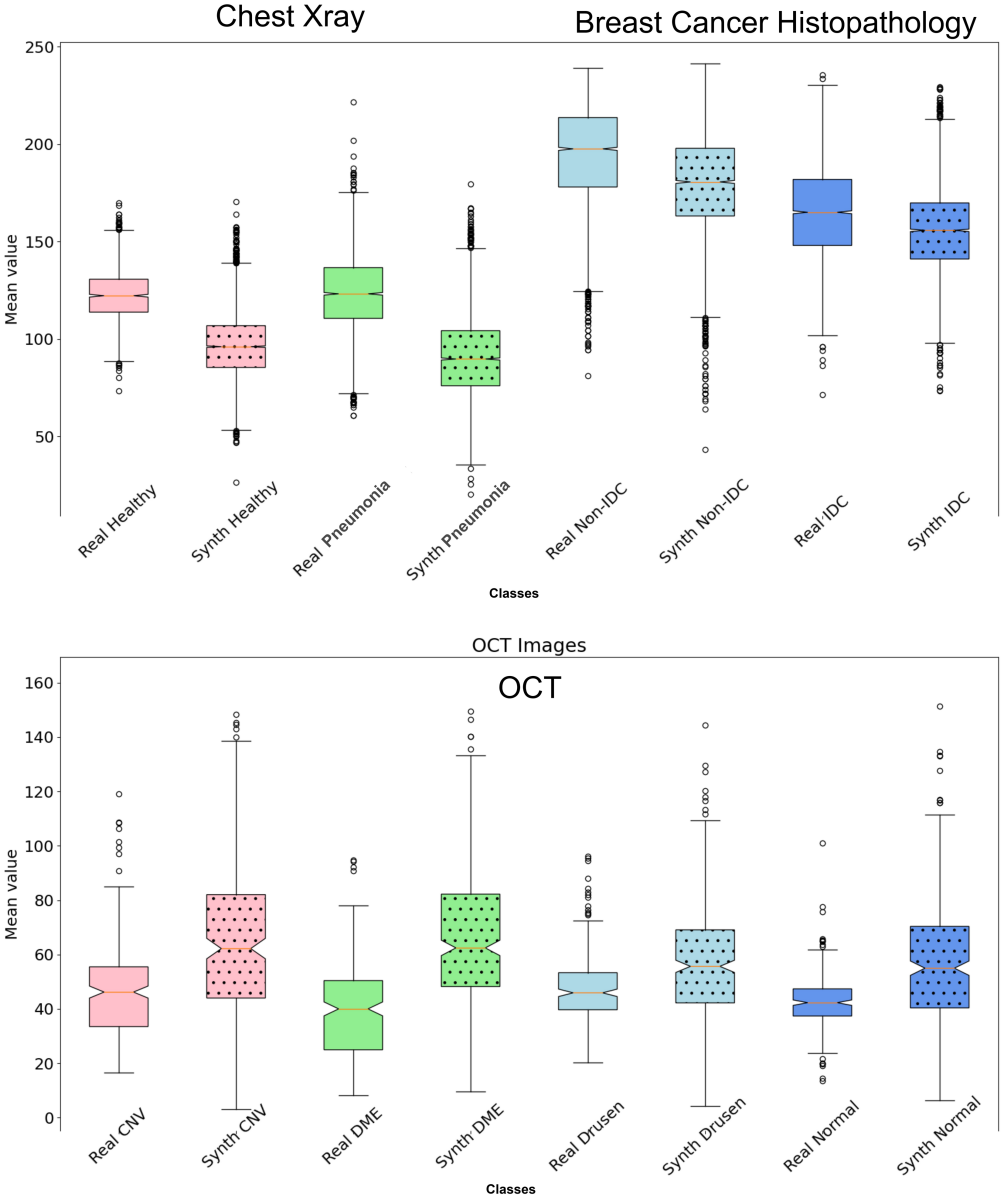
Box plot illustrating the mean values of the synthetic dataset compared to the real dataset for each class. There is no statistically significant difference in mean pixel values between the real and synthetic images across all datasets (*p* > 0.05 after FDR correction).

In the context of feature comparison, Figure 4 illustrates the t-SNE visualization of feature distributions for synthetic images generated by DDPM. For the OCT dataset, all abnormal classes (CNV, DME, and Drusen) were combined into a single ‘unhealthy’ class, while the ‘normal’ class was designated as the ‘healthy’ class. T-SNE was subsequently applied to this grouped dataset. In Figure 5, box plots illustrating the mean pixel values of real and synthetic data for each class of each dataset separately are presented. There was no statistically significant difference between mean pixel values from the real and synthetic images across all datasets and suggesting consistent performance across all datasets, with *p*-values of 0.073, (0.91 after FDR correction), and 0.11 for Chest X-ray, OCT, and Breast Cancer Histopathology, respectively.

### 3.4 Performance comparison of classifiers trained on real vs. synthetic medical images

Two distinct classifiers were developed and trained: one exclusively utilizing synthetic data and the other employing authentic data. Subsequently, both classifiers underwent evaluation using real test data previously unseen by the models. The comparative accuracy of these classifiers is presented in Table 1. While the classifier trained on real data exhibited superior performance relative to its synthetic counterpart across all evaluated cases, the observed discrepancies were found to be minimal and statistically non-significant. A comprehensive statistical analysis confirmed the similarity in performance metrics between the classifiers, suggesting a consistent performance trend across diverse datasets. The p-values for the chest X-ray, OCT, and breast cancer classification datasets, when comparing the F1 score, accuracy, and area under the curve (AUC), are 0.0705, 0.1903, and 0.201, respectively (*p* > 0.05; after FDR correction). These findings underscore a robust and consistent performance of the classifiers, independent of the data source—synthetic or real—in their training.

**Table 1:** Quantitative assessment of classification performance.

	Classifier	Precision	Recall	F1	AUC	Accuracy
Chest X-Ray Dataset	Synthetic	0.93	0.94	0.94	0.98	0.95
	Real	0.94	0.95	0.94	0.99	0.96
Breast Cancer Dataset	Synthetic	0.82	0.82	0.82	0.82	0.82
	Real	0.83	0.83	0.83	0.83	0.83
OCT Dataset	Synthetic	0.96	0.96	0.96	0.99	0.96
	Real	0.99	0.99	0.99	0.99	0.99

The table displays the outcomes of the classification model network trained on real data and DDPM-generated data for both the Chest X-ray dataset and Histopathology dataset (results are reported to two significant digits).

## 4 Discussion

The scarcity of adequate medical data poses a significant challenge in training AI and DL algorithms. The utilization of synthetic images while preserving important biomarkers not only alleviates the challenges of data collection and processing but also improves the scalability and accessibility of DL applications. In this work, we assessed and explored the effectiveness of state-of-the-art diffusion-based generative algorithm in synthesizing privacy-preserving medical data that can capture essential imaging biomarkers across multiple modalities, and our findings shed light on the fidelity and utility of these synthetic images.

Our results demonstrate that the synthesized medical images closely resemble real images, both qualitatively and quantitatively. Visual inspection revealed that the diffusion model accurately captured key biomarkers indicative of various medical conditions across different modalities. The sensible IS, FID, and nearest-SSIM values underscore the efficacy of DDPM in generating high-fidelity synthetic images that faithfully represent real-world medical data.

The ability of the diffusion model to faithfully preserve biomarkers such as lung markings in Chest X-ray images, retinal abnormalities in OCT images, and histopathological features in breast cancer images is particularly promising. This preservation of critical biomarkers is crucial for ensuring the clinical relevance and utility of synthetic images in various healthcare applications. Furthermore, by analyzing the distribution of mean pixel values of the histopathological images as in Figure 5, it is apparent that the synthetic dataset closely resembles the pattern observed in the real dataset. Regarding the OCT images, while the mean pixel values exhibit proximity, the synthetic dataset is characterized by a larger standard deviation specifically for Drusen and Normal class. Conversely, the real dataset displays smaller standard deviations across nearly all four classes in comparison to the synthetic dataset. However, there is no statistically significant difference in mean pixel values between the real and synthetic images across all datasets (*p* > 0.05 after FDR correction).

In the classification assessment, the classifier trained with synthetic data exhibited slightly inferior performance compared to the model trained on real data, yet their performance was comparable (p > 0.05 after FDR correction). Our evaluation indicated that training classification networks with synthetic images yields promising results, with F1 score and AUC reaching levels of 0.8–0.99 compared to when trained with real images. Interestingly, despite the high FID score for histopathological images, the classifier successfully separated IDC/No-IDC with comparable performance to the model trained on real data.

In addition, the classifier successfully identified medical biomarkers within the synthetic OCT images despite utilizing a dataset size ten times smaller. It achieved performance comparable to that of the classifier trained on real data, which benefited from a total of 84,000 images, with nearly 8,000 images per class. These results suggest that synthetic images generated by DDPM can serve as effective substitutes for real data in classification tasks, offering a viable solution for addressing data scarcity and privacy concerns in medical imaging research. It is important to note that these issues must be handled with caution and thoroughly validated, especially in clinical contexts.

Although we only assessed synthetic data for classification tasks, we expect the applicability to other tasks such as segmentation and object detection. It is worth noting that our goal was not to determine the effectiveness of diffusion-based models merely for classification but rather to explore whether synthetic data can preserve essential imaging biomarkers. Although each dataset in our analysis comes from a single source, the developed methodology could be broadly applied to train classifiers using data from multiple sources. This approach could generate more diverse synthetic datasets for identifying diseases in images from different institutions. Despite our efforts to address this issue, the challenge remains in obtaining similar datasets with aligned purposes (similar disease patterns) from multiple institutions.

It should be noted that the primary objective of our work was not to identify or benchmark the best generative model for synthetic image generation but rather to explore the feasibility of fully synthetic datasets generated using DDPMs in preserving critical medical biomarkers for downstream tasks. Diffusion models, such as DDPMs, have recently demonstrated impressive results in generative tasks across various domains, motivating us to examine their potential within medical imaging modalities. Specifically, we focused on whether DDPM-generated synthetic datasets could maintain diagnostic relevance, particularly in preserving hidden biomarkers. Although a direct comparison with other GAN-based approaches could strengthen the evaluation, we chose to limit our investigation to DDPMs, given the focused scope of this study, enabling a detailed analysis of their capabilities.

In conclusion, our study demonstrates the feasibility and utility of generating synthetic medical images using DDPM while preserving important imaging biomarkers. These synthetic images hold promise for advancing various applications in healthcare, from AI algorithm development to data augmentation and privacy-preserving data sharing. By bridging the gap between synthetic and real-world medical imaging data, our approach paves the way for future innovations in medical imaging research and clinical practice.

## Data Availability

The datasets used for pretraining the diffusion models, as presented in the study, are included within the article. Additionally, all generated datasets are publicly available at https://github.com/serag-ai/Multimodal-Synthetic-Medical-Images. For further inquiries, please contact the corresponding author.
